# Réaction sclérale pseudotumorale au fil de polyester téréphtalique tressé et aux cils pris accidentellement après chirurgie de décollement de rétine en ab-externo

**DOI:** 10.11604/pamj.2016.25.101.8980

**Published:** 2016-10-20

**Authors:** Sophia El Hamichi, Abdelbarre Oubaaz

**Affiliations:** 1 Military Hospital Mohammed V of Rabat, Rabat, Morocco

**Keywords:** Reaction pseudotumorale, decollement de retine, chirurgie ab-externo, Pseudotumoral reaction, retinal detachment, ab-externo surgery

## Image en médecine

Il s'agit d'une réaction pseudotumorale aux cils et au fil de MERSUTURE+ survenue plusieurs mois après chirurgie de décollement de rétine, chez un patient de 50 ans. Les cils ont été pris accidentellement et par inattention du chirurgien opérateur. Le résultat était confirmé a l'examen anatomopathologique.

**Figure 1:**
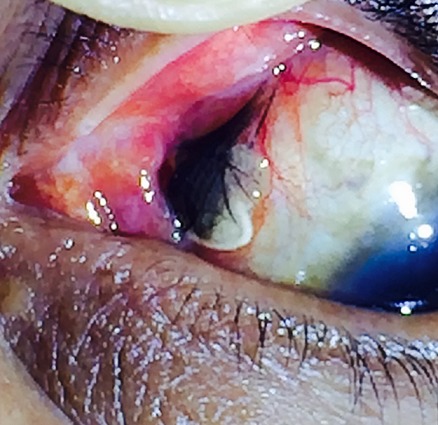
Réaction pseudotumorale au fil de MERSUTURE+ et aux cils

